# An Audit on Substance Misuse Screening and Documentation in Acute Inpatient General Psychiatry Ward

**DOI:** 10.1192/bjo.2025.10556

**Published:** 2025-06-20

**Authors:** Ariana Axiaq, Ly Bui Dieu, Joseph Itimi

**Affiliations:** 1 University of Edinburgh, Edinburgh, United Kingdom; 2 St John’s Hospital, Livingston, United Kingdom

## Abstract

**Aims:** Scotland has the highest drug-related death rate in Europe, with 27.7 deaths per 100,000 people. The average age of drug-related deaths in Scotland has increased from 32 in 2000 to 44 in 2021, with a significant proportion involving multiple substances. According to NICE and Scottish Intercollegiate Guidelines Network (SIGN) guidelines, best practice management for inpatients with co-occurring substance misuse and mental health should involve: Screening and Identification of Substance Misuse, Integrated Dual Diagnosis Care, Pharmacological and Psychosocial Interventions, Harm Reduction Approaches in Acute Psychiatric Wards and Safe Discharge and Aftercare Planning. We have designed a closed-audit loop which has evolved into a quality improvement project to enhance the identification, management, and follow-up care of substance misuse among inpatients in general psychiatry ward at St John’s Hospital, Livingston, Scotland. This project seeks to achieve an 80% compliance rate in substance use assessments, implement standardized protocols for managing active substance misuse, and develop a policy for handling patients under the influence, in possession, or distributing substances by December 2025. Data presented in this abstract is for the closed-loop audit part of a larger quality improvement project.

**Methods:** Data for the initial audit was collected from Trak, the electronic patient record (EPR) system, for patients admitted to and discharged from a mixed acute general adult ward at St John’s Hospital, during the period from 13 February to 13 May 2024. Data was collected on documentation of previous substance misuse and substance misuse urine screening. As an intervention, our team introduced staff reminders for biological testing and incentivised staff, patients and carers to provide feedback on substance misuse history, substance misuse on the ward and testing. From 30 October till 30 January, a further cycle was conducted to assess for changes post-intervention.

**Results:** The closed-loop audit included 84 patients pre-intervention and 49 patients post-intervention. 35.3% had co-morbid schizophrenia/psychosis and a further 24.7% had combined depressive disorder and suicidality. Alcohol misuse was the highest reported substance misuse (33.33%), followed by cannabis (27.03%) and cocaine (11.71%). Post-intervention there was a 35.18% increase in biological testing compliance (post-intervention 57%) and 51.36% increase in substance misuse history documentation compliance (post-intervention 75%).

**Conclusion:** Substance misuse worsens prognosis for many patients with other mental health co-morbidities. We have identified that compliance is still low for documentation and testing. By identifying gaps in achieving compliance, this project seeks to guide better local policy in order to implement evidence-based practices improving patient outcomes.

